# Identification of Rice Accessions Having Cold Tolerance at the Seedling Stage and Development of Novel Genotypic Assays for Predicting Cold Tolerance

**DOI:** 10.3390/plants12010215

**Published:** 2023-01-03

**Authors:** Qi Yongbin, Patcharaporn Summat, Natjaree Panyawut, Kannika Sikaewtung, Khanittha Ditthab, Keasinee Tongmark, Sriprapai Chakhonkaen, Numphet Sangarwut, Thiwawan Wasinanon, Kanokwan Kaewmungkun, Amorntip Muangprom

**Affiliations:** 1 Institute of Crop Science and Nuclear Technology Utilization, Zhejiang Academy of Agricultural Sciences, Hangzhou 310021, China; 2 National Center for Genetic Engineering and Biotechnology, Thailand Science Park, Khlong Luang, Pathum Thani 12120, Thailand; 3 Department of Biotechnology, Faculty of Science and Technology, Thammasat University, Rangsit Centre, Khlong Luang, Pathum Thani 12120, Thailand; 4 Nadi District Agricultural Extension Office, Chamanan Road, Nadi Subdistrict, Nadi District, Prachinburi 25220, Thailand

**Keywords:** cold tolerance, rice germplasm, functional markers, *NAC*, *COLD1*, *LOC_Os10g34840*

## Abstract

Rice is susceptible to cold stress at the seedling stage, which can delay growth and decrease yield. We evaluated 187 rice accessions for cold tolerance at the seedling stage and developed genotypic assays for three markers. All *japonica* (20/20) and 20/140 *indica* accessions were highly cold tolerant. Two SNP markers specific for *COLD1* and *LOC_Os10g34840* were practical to use by normal agarose gel. The SNP marker specific for *COLD1* was highly specific for predicting cold tolerance. However, the sensitivity of this marker was low as several cold-tolerant *indica* accessions lacked the cold-tolerant allele. The *LOC_Os10g34840* marker was slightly more sensitive than the *COLD1* marker for predicting highly cold-tolerant accessions. An insertion/deletion variant in the *NAC6* gene was identified as a novel cold tolerance marker. The *NAC6* marker predicted more highly cold-tolerant accessions compared with the other two markers. The SNP marker specific for *LOC_Os10g34840* and the *NAC6* marker were present in several tested subgroups, suggesting their wide effects and distribution. The three markers combined predicted the most highly cold-tolerant accessions, indicating that the marker combination is superior for applications such as marker-assisted breeding. The cold-tolerant accessions and the genotypic marker assays will be useful for future rice breeding.

## 1. Introduction

Rice is a staple food for half of the world’s population and is cultivated in more than 100 countries. However, rice is susceptible to low temperature stress, which affects growth, productivity, quality formation, and geographical distribution of rice plants [1,2]. Cold stress at the seedling stage can cause chlorosis, necrosis, reduction in vigor, and delay in seedling emergence leading to delay crop maturation. Together, these effects reduce crop yield [3]. In addition, cold stress affects not only the yield but also the quality of rice [4]. Cold stress is an important problem for rice cultivation, even in the major rice-producing Lower Mekong tropical countries (Thailand, Cambodia, Laos, Burma, and Vietnam) [5].

Asian cultivated rice (*Oryza sativa*) comprises two subspecies: *indica* and *japonica*. Through selective breeding over centuries, cultivated rice has acquired agricultural traits such as high grain yield and environmental tolerance. *Japonica* rice plants are generally more cold tolerant than *indica* cultivars [6,7,8]. *Japonica* rice plants harboring genetic variants conferring cold resistance can be bred with *indica* to develop cold-resistant varieties. However, cold tolerance is a complex trait controlled by multiple genes, which presents a challenge for developing new cold-resistant varieties. Quantitative trait loci (QTLs) mapping and genome-wide association study (GWAS) are two widely used tools for identifying the genetic control of complex traits.

Genetic loci controlling cold tolerance in rice have been discovered by QTL mapping using bi-parental populations [6,9]. However, bi-parental mapping has the major drawback due to the limitation of genetic background to parental lines. Recently, GWAS has also been used for cold tolerance in rice, with the advantage of using a large number of accessions for genetic loci controlling the trait. In addition, microarray and RNA sequencing technology were used for genome-wide gene expression in rice under cold stress [10,11,12]. The genes obtained from these studies could be useful for further studies on mechanisms controlling cold tolerance, and development of molecular markers used for selection of cold tolerant rice plants.

Several QTLs and genes responsible for cold tolerance have been identified [13]. The cold tolerant gene and QTL are useful for the development of molecular markers used for facilitating breeding of cold tolerance in rice [6,14]. Because it is difficult to identify plants having genes responsible for cold tolerance using phenotypes, marker-assisted selection is a valuable method for developing cold-tolerant cultivars.

The NAC (NAM, ATAF and CUC) superfamily is one of the largest plant-specific transcription factor families. NAC transcription factors are involved in a wide range of abiotic and biotic stress responses including cold stress [15]. *OsNAC6* was reported as a transcriptional activator in response to abiotic and biotic stresses, and this gene could be useful for the improvement of stress tolerance in plants [16]. *OsNAC6* was reported to mediate root structure and enhance drought tolerance [17]. In addition, expression of *OsNAC6* was shown to be increased under cold stress [12,18].

Using recombinant inbred lines (RILs) generated from a cross between chilling-tolerant Nipponbare (*japonica*) and chilling-sensitive 93-11 (*indica*) cultivars, Ma et al. [6] identified the QTL locus, *COLD1*, conferring chilling tolerance in *japonica* rice. *COLD1* encodes a regulator of G-protein signaling, and it interacts with the G-protein to activate the Ca^2+^ channel for sensing low temperature. A nonsynonymous single-nucleotide polymorphism (SNP) in the *COLD1* fourth exon was found to distinguish all cold-tolerant from cold-sensitive accessions. Moreover, evolutionary analysis suggests that this variant was selected during the domestication of *japonica* rice. In addition, overexpression of *COLD1* jap significantly increases chilling tolerance. These finding are of great potential for rice molecular breeding.

Using GWAS with a 1033 rice accession diversity panel, *LOC_Os10g34840* was identified as the candidate gene for cold tolerance in rice at the seedling stage, and SNP2G was identified to be responsible for cold tolerance at the seedling stage. This SNP is a nonsynonymous variant predicted to affect the protein folding and function of a putative pectin lyase. The cold-tolerant allele, SNP2G, was reported to be present in 80.08% in temperate *japonica* but only 3.8% of *indica*. The SNP2G mutation site was used to design a cleaved-amplified polymorphic sequence (CAPS) marker, which was used as marker-assisted selection (MAS). Most of near-isogenic lines (NILs) having SNP2G showed better growth and significantly higher survival rates than those of the NILs having SNP2A [14]. The authors hoped that cold tolerant loci and the functional markers will be useful for breeding cold-tolerant varieties and for studying the molecular basis of cold tolerance in rice.

Because cold tolerance is a very complex trait controlled by multiple genes, it is very difficult for breeders to select desirable plants due to the lack of discrete phenotypes. In addition, this trait is affected by the environment. Functional markers or gene markers are developed from DNA polymorphisms within the genes that cause phenotypic trait variations [19]. Functional markers are directly linked to the allele of the target traits [20]. Thus, for marker-assisted selection (MAS), these markers are better than random DNA markers such as simple sequence repeat (SSR) and single nucleotide polymorphism (SNP). The *COLD1* and *LOC_Os10g34840* functional markers may be insufficient to accurately predict cold tolerance; hence, more markers are required. In addition to genetic mapping methods, genes potentially controlling cold tolerance in rice can be identified using comparative transcriptomic methods, such as microarray and RNA sequencing [11,12,21].

Polymorphisms in *COLD1*, *LOC_Os10g34840*, and *OsNAC6* can be used for the development of practical gene markers used as MAS for breeding of cold-tolerant plants in a breeding laboratory having limited resources. Although a CAPS marker was developed for a cold-tolerant allele of *LOC_Os10g34840*, a practical marker without the need for restriction enzyme digestion could be an alternative method. In addition, to our knowledge, there is no report of a *NAC6* marker applied to MAS associated with cold tolerance based on differential expression in transcriptomic studies.

This work evaluated rice germplasms for cold tolerance at the seedling stage, and developed functional markers from genes conferring cold tolerance, *COLD1*, and *LOC_Os10g34840*. These two gene markers were developed to detect polymorphic SNPs using normal agarose gel. In addition, a 222 bp deletion in the *OsNAC6* gene was identified as a novel marker of cold tolerance among the accessions. The germplasms and the markers reported in this study will be useful for future rice breeding.

## 2. Results

### 2.1. Phenotypic Variation in Response to Cold Treatment

**Figure 1:**
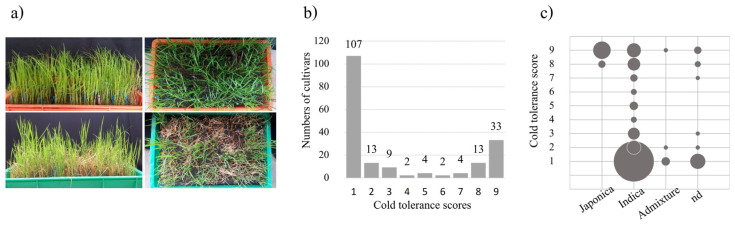
Phenotypic variation of rice accessions in response to cold treatment. (**a**) Top, 10-day old seedlings before cold treatment; bottom, 10-day old seedlings after cold treatment at 6 ± 2 °C for three days and recovery at 30 °C for 10 days. (**b**) Phenotypic variation of rice accessions in response to cold treatment, X-axis is cold tolerant score (1–9), Y-axis is number of rice accessions. (**c**) The cold tolerance score structure (Y-axis) in different subspecies (X-axis).

To assess the phenotypic variation in cold tolerance, 10-day old rice seedlings (at three-leaf stage) were evaluated. A total of 187 rice accessions were tested in this study, of which 20 were *japonica*, 140 were *indica*, 6 were admixture, and 21 were not determined (nd) (Table S1). Phenotypic variation among 187 rice accessions in response to cold treatment at the seedling stage was observed. Rice seedlings aged 10 days before (top) and after cold treatment (bottom) are shown in Figure 1a. Phenotypic variation of the rice accessions in response to cold treatment is shown in Figure 1b. The cold tolerant scores of each accession are listed in Table S1. Approximately 68.9% (129/187) of these accessions were highly sensitive (score 1–3) and approximately 24.5% (46/187) were highly tolerant (score 8–9) (Figure 1b, Table S1). The highly sensitive accessions were *indica* and admixture. All of the tested *japonica* (20/20) and 20 out of 140 tested *indica* were highly tolerant (Figure 1c).

### 2.2. Marker Development and Genotyping

#### 2.2.1. NAC6

**Figure 2:**
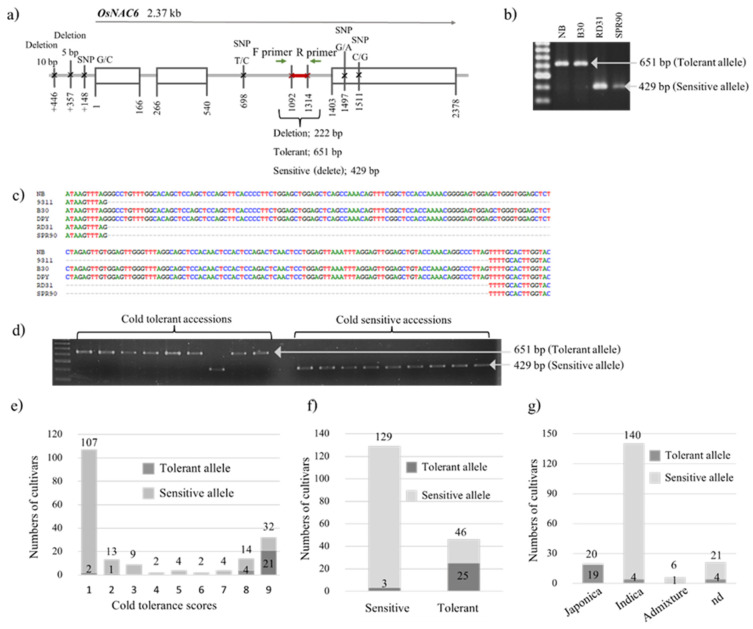
Mutation in *NAC6* and its marker used for genotyping. (**a**) Structure of *NAC6* showing polymorphic sequences between *japonica* (Nipponbare) and *indica* (9311). (**b**) Validation of the genotypic assay for two cold-tolerant (NB and B30) and two cold-sensitive (RD31 and SPR90) reference cultivars. (**c**) Sequence alignment of amplicons from cold-tolerant (NB, DPY, and B30) and cold-sensitive (RD31 and SPR90) reference cultivars. (**d**) Representative examples of genotyping ten cultivars scored as cold-sensitive and ten cultivars scored as cold-tolerant among 187 accessions. (**e**) Allele frequency of *NAC6* distributed in 1–9 cold tolerant scores of 187 rice accessions. (**f**) Allele frequency of *NAC6* in extremely tolerant (score 8,9) and extremely sensitive (score 1–3) rice accessions. (**g**) The distribution of tolerant and sensitive alleles of *NAC6* in each subspecies group.

To seek additional markers of cold tolerance in other genes apart from *COLD1* and *LOC_Os10g34840*, genomic sequence putatively involved in cold tolerance, 2.37 kb DNA sequences of *NAC6* from Nipponbare, were compared with those of 93–11. Several DNA polymorphisms between them were identified (Figure 2a). The deletion of 222 bp in the 2nd intron was of interest because this polymorphism can be used for development of an indel marker, providing DNA patterns easy to observe by normal agarose gel. To confirm this polymorphic site, the sequences flanking this mutation were used to design primers to genotype two cold-tolerant cultivars, DPY and B30, and two cold-sensitive cultivars, SPR90 and RD 31, resulting in polymorphic bands easily observed (Figure 2b). These PCR bands were sent for sequencing. The results showed that the sensitive cultivars contained 222 bp deletion compared with the corresponding sequence of the tolerant cultivars (Figure 2c). Then, this marker was used to genotype the 187 rice accessions to evaluate the allele frequency of this gene. Samples of the genotyping are shown in Figure 2d. The results of genotyping demonstrating the distribution of tolerant and sensitive alleles in each cold tolerant score are shown in Figure 2e. The analysis showed that 54.4% (25 out of 46) of highly tolerant accessions (score 8 and 9) had the tolerant allele, and 97.7% (126 out of 129) of highly sensitive accessions (score 1–3) had the sensitive allele (Figure 2f). In addition, the results from genotyping showed that the tolerant allele was presented in all subspecies.

#### 2.2.2. COLD1

**Figure 3:**
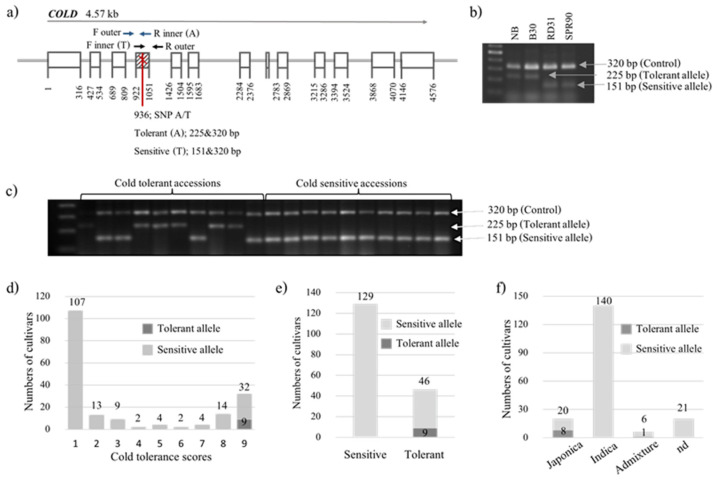
Mutation in *COLD1* and its marker used for genotyping. (**a**) Structure of *COLD1* showing polymorphic sequences between *japonica* (Nipponbare) and *indica* (9311). (**b**) Validation of the genotypic assay for two cold-tolerant (NB and B30) and two cold-sensitive (RD31 and SPR90) reference cultivars. (**c**) Representative examples of genotyping ten cultivars scored as cold-sensitive and ten cultivars scored as cold-tolerant among 187 accessions. (**d**) Allele frequency of *COLD1* distributed in 1–9 cold tolerant scores of 187 rice accessions. (**e**) Allele frequency of *COLD1* in extremely tolerant (score 8,9) and extremely sensitive (score 1–3) rice accessions. (**f**) The distribution of tolerant and sensitive alleles of *COLD1* in each subspecies group.

A single-nucleotide mutation (A/T) in *COLD1* was reported to confer cold tolerance at the seedling stage (Figure 3a; [6]). This mutation was used to design SNP marker using two pairs of primers in a single PCR tube simultaneously amplifying both mutant and normal alleles as well as an internal control band. To confirm this polymorphic site, the sequences flanking this mutation and the mutation site were used to design primers and genotyped two cold-tolerant cultivars, DPY and B30, and two cold-sensitive cultivars, SPR90 and RD 31. The results showed clearly polymorphic bands between tolerant and sensitive cultivars easily observed by 2% normal agarose gel (Figure 3b). Then, this marker was used to genotype the 187 rice accessions to evaluate the allele frequency of this gene. Samples of the genotyping are shown in Figure 3c. The results of genotyping demonstrating the distribution of tolerant and sensitive alleles in each cold tolerant score are shown in Figure 3d. The analysis showed that 19.6% (9 out of 46) highly tolerant accessions (score 8 and 9) had the tolerant allele, and 100% (129 out of 129) highly sensitive accessions (score 1–3) had the sensitive allele (Figure 3e). In addition, the result of genotyping showed that the tolerant allele of *COLD1* was found mainly in *japonica* type (Figure 3f).

#### 2.2.3. LOC_Os10g34840

**Figure 4:**
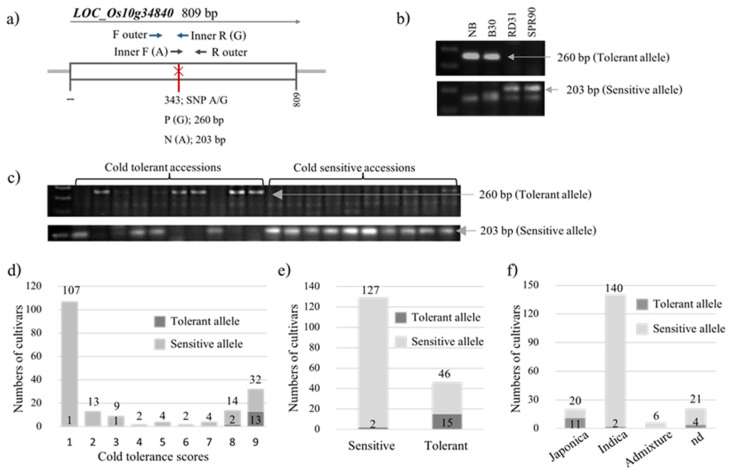
Mutation in *LOC_Os10g34840* and its marker used for genotyping. (**a**) Structure of *LOC_Os10g34840* showing polymorphic sequences between *japonica* (Nipponbare) and *indica* (9311). (**b**) Validation of the genotypic assay for two cold-tolerant (NB and B30) and two cold-sensitive (RD31 and SPR90) reference cultivars. (**c**) Representative examples of genotyping ten cultivars scored as cold-sensitive and ten cultivars scored as cold-tolerant among 187 accessions. (**d**) Allele frequency of *LOC_Os10g34840* distributed in 1–9 cold tolerant scores of 187 rice accessions. (**e**) Allele frequency of *LOC_Os10g34840* in extremely tolerant (score 8,9) and extremely sensitive (score 1–3) rice accessions. (**f**) The distribution of tolerant and sensitive alleles of *LOC_Os10g34840* in each subspecies group among 187 accessions.

*LOC_Os10g34840* was reported to control cold tolerance at the seedling stage due to a single-nucleotide mutation (G/A) (Figure 4a, [14]). This mutation was used to design a SNP marker using two pairs of primers for allele-specific amplifications. To confirm this polymorphic site, the sequences flanking this mutation and the mutation site were used to design primers and genotyped two cold-tolerant cultivars, DPY and B30, and two cold-sensitive cultivars, SPR90 and RD31. The results showed clearly polymorphic bands between tolerant and sensitive cultivars easily observed by 2% normal agarose gel (Figure 4b). Then, this marker was used to genotype the 187 rice accessions to evaluate the allele frequency of this gene. Samples of the genotyping are shown in Figure 4c. The results of genotyping demonstrating the distribution of tolerant and sensitive alleles in each cold tolerant score are shown in Figure 4d. The analysis showed that 32.6% (15 out of 46) of highly tolerant accessions (score 8 and 9) had the tolerant allele, and 98.4% (127 out of 129) of highly sensitive accessions (score 1–3) had the sensitive allele (Figure 4e). In addition, the result of genotyping showed that the tolerant allele of *LOC_Os10g34840* was found in both *japonica* and *indica* types (Figure 4f).

#### 2.2.4. Marker Combination for Predicting Cold Tolerance

**Figure 5:**
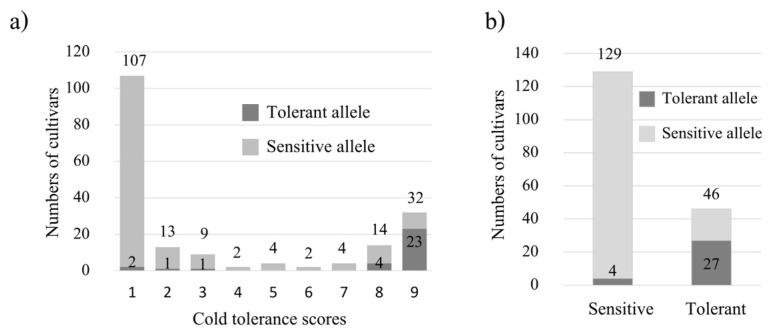
Genotyping using the three gene-specific markers and prediction of cold tolerance. (**a**) Allele frequency of the presence of one or more cold-tolerant allele from the three loci distributed in 1–9 cold tolerant scores of 187 rice accessions. (**b**) Allele frequency of the presence of one or more cold-tolerant allele from the three loci in extremely tolerant (score 8,9) and extremely sensitive (score 1–3) rice accessions.

Since cold tolerance is a complex trait, we wondered if the combination of markers would be a better predictor of cold tolerance than single markers. The accessions were separated into two groups according to the presence of one or more cold-tolerant allele from the three loci versus homozygous cold-sensitive allele for all three loci. Using all three markers together, the result is shown in Figure 5a. The prediction of highly cold-tolerant accessions was slightly more sensitive using the combined markers (27 out of 46) compared with the best single marker *NAC6* (25 out of 46) (Figure 5b).

## 3. Discussion

Germplasm is a highly valuable source used for the development of new varieties with desirable traits. The identified accessions with high cold tolerance will be useful for the development of new varieties having better cold tolerance. These varieties can be used to improve rice production affected by low temperatures. Most of rice accessions tested in this study were *indica*, although most of them were upland rice. This was different from a study indicating that *indica* genotypes are predominantly adapted to the lowland, while *japonica* rice is more adapted to the upland and highland regions [22]. The cold tolerance phenotype was assessed among the 187 rice accessions, of which only a few were highly cold tolerant (Figure 1; Table S1), probably because most of them were of the *indica* type, which in general is more cold sensitive than *japonica* [14,23,24]. All of the tested *japonica* accessions were cold tolerant. Interestingly, several of our improved *indica* varieties showed cold tolerance.

The gene-specific markers developed in this study, particularly for SNP detection, are practical to use by normal agarose gel. In general, sequencing or other laborious techniques need to be used for SNP detection [25,26,27,28]. Our SNP gene-specific markers can be used effectively in small breeding laboratories having only a set of equipment needed for normal PCR. In addition, our Indel gene-specific marker for *NAC6* yielded PCR products with more than 200 bp size difference, easily observed by normal PCR and agarose gel.

Results from sequence alignment of *NAC6* genes from the *indica* and *japonica* cultivars showed several polymorphic sites (Figure 2). Most of them were SNP or short deletions (≤10 bp), which were not easy to observe by normal agarose gel. However, there was a big deletion of 222 bp in the *indica* cultivars resulting in a polymorphic site easily observed. Conserved sequences flanking the polymorphic site were used for marker development. Using this marker to genotype the 187 rice accessions, the results showed that it can detect the highest number of resistant accessions compared to the other two markers developed in this study. In addition, 90% of *japonica* accessions (18/20) extremely tolerant to cold stress (score 8,9) have the tolerant allele for this gene, and 97.7% (126/129) extremely sensitive accessions (score 1,2,3) have the sensitive allele, similar to previous study reporting using indel markers developed from *LOC_Os03g09140* (*Osryh1*) to genotype 153 rice accessions [23]. In addition, this marker could detect two extremely tolerant *indica* accessions, indicating that the tolerant allele of this gene was present in both *japonica* and *indica* types. Furthermore, the tolerant allele of this gene was present also in an admixture type and some of the not determined group, suggesting wide distribution and effects of this allele in various types of rice. Unlike *COLD1* and *LOC_Os10g34840*, polymorphism in *NAC6* associated with cold tolerance has not been reported. The *NAC6* gene indel marker is located in the second intron of the gene. Therefore, it is difficult to assess the effect of this variant on *NAC6* gene function. *NAC6* mRNA splicing or stability could be affected by the indel and have a consequent effect on *NAC6* protein levels. Alternatively, the *NAC6* variants could be closely linked to another gene with variants that have a direct relationship to cold tolerance. To test whether the *NAC6* indel is a functional marker of cold tolerance, specific assays of *NAC6* gene function are required.

A single point mutation, SNP2 (A/T), in *COLD1*, is associated with cold tolerance, A in *Japonica* to T/C in *indica* and in O. nivara. However, C was detected only in a small number of tested accessions (7.8%, 10/127). Most of them were rice accessions from India [6]. In our study, the SNP marker for *COLD1* was developed as an allele-specific marker, A for tolerant and T for sensitive types. This marker is practical to use because the polymorphism is easily observable by normal agarose gel used in the routine PCR laboratory, and no other sophisticated equipment is needed. In addition, both wild type and mutant alleles can be amplified in a single PCR tube, resulting in cost and time reduction compared to allele-specific amplification in separated PCR tubes. Furthermore, the polymorphism can be observed without restriction enzyme digestion, different from allele-specific markers previously reported for Pi2 [29]. Results from genotyping using this *COLD1* marker indicated that only a few accessions (9/187) of this set of rice germplasm had a tolerant allele. However, this marker is highly specific because all accessions having a tolerant allele show extremely tolerant phenotype and all extremely sensitive accessions had a sensitive allele. Interestingly, none of extremely tolerant *indica* had a tolerant allele of this gene.

In this study, an allele-specific marker for *LOC_Os10g34840* was developed based on G/A mutation (SNP2; G/A). *LOC_Os10g34840* was reported as a functional gene controlling cold tolerance at the seedling stage, and SNP2^G^ is a cold-tolerant allele. SNP2^A^ allele was reported to present mainly in accessions from low altitudes, where most of the accessions are *indica* and tropical *japonica* types, while the SNP2^G^ allele was found in higher altitudes [14]. Xiao et al. [14] developed the cleaved amplified polymorphic sequence (CAPS) marker to detect this point mutation and used as marker-assisted selection (MAS) to genotype near-isogenic lines (NIL) generated from cold-sensitive *indica* (Nanjing11) and cold-tolerant *japonica* (Nipponbare). The results showed that NIL2^G^ plants demonstrated significantly better cold tolerance than those of the NIL2^A^ in terms of survival rates at seedling stage and 1000-seed weights [14]. However, their marker requires restriction enzyme digestion analysis. Our SNP allele-specific marker for *LOC_Os10g34840* developed in this study did not need digestion. This marker showed that 32.6% (15 out of 46) of highly tolerant accessions (score 8 and 9) had the tolerant allele. The highly tolerant accessions included *japonica*, *indica*, and the not determined group, suggesting that this tolerant allele is widely distributed. In addition, this marker is highly specific for detecting the highly sensitive allele (98.4%, 127 out of 129).

The *COLD1* marker is the least sensitive, but most specific for predicting highly cold-tolerant accessions (Table S1). The *LOC_Os10g34840* marker was slightly more sensitive than the *COLD1* marker for predicting highly cold-tolerant accessions. The *NAC6* gene indel marker was the most sensitive marker for predicting highly cold-tolerant accessions. Importantly, the *NAC6* marker could predict highly tolerant *indica* accessions which lacked cold-tolerant alleles for the other gene tested (Table S1). Several of the Thai *indica* varieties were highly cold tolerant but did not possess cold-tolerant marker alleles (Table S1), suggesting that other markers are needed for robust prediction of cold tolerance in *indica* varieties.

Because cold tolerance is controlled by multiple genes, using several markers developed from several genes could help to identify more cold-tolerant accessions having different tolerant alleles. Accordingly, using three gene-specific markers in this study could identify 58.7% (27 out of 46) of highly tolerant accessions, instead of using individual marker for *NAC6* (54.4%), *COLD1* (19.6%), or *LOC_Os10g34840* (32.6%). Our results suggested that if more cold tolerance genes were used, this proportion might be increased. However, there is insufficient information to determine the additive and non-additive (dominance and epistasis) effects among these markers on the cold tolerance prediction accuracy, since markers in other genes also control the cold tolerance phenotype. Therefore, if accessions were genotyped for more markers from other genes, the cold tolerance prediction accuracy could be improved.

## 4. Materials and Methods

### 4.1. Plant Materials

For cold treatment, a total of 187 rice accessions including Thai and exotic rice genotypes (Supplementary Table S1) were used for phenotyping and genotyping. The Thai accessions included upland and lowland rice genotypes collected nationwide, which were obtained from Genebank (Department of Agriculture, National Plant Genetic Resources Center, Thailand) and Thai farmers. The exotic rice accessions were obtained from the International Rice Research Institute (IRRI). The details of each accession are described in Table S1. The majority of the accessions used in this study comprise upland rice, with a tendency/expectation to have better cold tolerance than the lowland *indica* cultivars. The *indica* cultivars and the exotic rice lines were used in this study because they have several other desirable agronomic traits, which could be useful for the development of new cold-resistant varieties with better traits.

Two cold-tolerant cultivars, DPY and B30, and two cold-sensitive cultivars, SPR90 and RD 31, were used for marker development.

About 20 seeds of each accession were germinated in Petri dishes in an incubator. The germinated seeds were planted into 2 × 2 wells in a 50-well tray and cultivated under natural conditions. When the plants were about 15 days old, young leaves from about 10 plants per accession were harvested and used for genotyping. Total genomic DNA was extracted using the standard CTAB extraction protocol [30].

### 4.2. Cold Treatment and Evaluation of Cold Tolerance

For each accession, 20–30 seeds were germinated in Petri dishes in an incubator. The germinated seeds were planted in 2 × 2 wells in a 50-well tray at 30 ± 2 °C. In each tray, Nipponbare variety seeds were planted in four wells with different locations in the tray, which were used as positive controls. When the plants were about 10 days old (three-leaf stage), the trays of seedlings were moved to air-conditioned glass rooms for cold treatment at 6 ± 2 °C, with a 12 h day length of 126 µmol m^−2^ s^−1^ light intensity and a relative humidity of 75 to 85% for three days. Then, the seedlings were transferred back to 30 °C. After 10 days at 30 °C, the seedlings were scored for cold tolerance. The experiment was repeated three times under the same conditions. The seedlings were scored for cold tolerance using survival rate (calculated as the number of living plants/number of total plants × 100) on a 1–9 scale. For scores of 1, 2, 3, 4, 5, 6, 7, 8, and 9, the survival rate is ≤20, between 21 and 30, between 31 and 40, between 41 and 50, between 51 and 60, between 61 and 70, between 71 and 80, between 81 and 90, and >90, respectively.

### 4.3. Marker Development and Genotyping

#### 4.3.1. NAC6 (LOC_Os01g66120)

Because *japonica* and *indica* showed different levels of cold tolerance, differences in *NAC6* gene sequences between these two subspecies could be involved in this process. Sequence alignment of *NAC6* gene of Nipponbare (*japonica*) and 93-11 (*indica*) was performed using Clustal W program. The 222 bp difference between them was designed to be an Indel marker using the conserved flanking sequences as primers (Table 1). The maker was used to amplify DNA sequences of two cold-tolerant and two cold-sensitive cultivars. The PCR products were sent for sequencing. Then, the marker was used to genotype 187 rice accessions. The PCR amplification was performed in 10 µL of reaction mixture, containing 1 µL of 10× Taq DNA polymerase buffer, 1 µL of 2 mM dNTP, 0.4 µL of 50 mM MgCl_2_, 0.1 µL of 5 U/µL Taq DNA polymerase (Invitrogen, Van Allen Way Carlsbad, CA, USA), 2 µL of DNA (10–50 ng/µL), 1 µL of 10 µM of forward or reverse primers. Thermal profiling was set with initial denaturation temperature of 95 °C for 5 min followed by 35 cycles of denaturation (95 °C for 30 s), annealing (59 °C for 30 s) and extension (72 °C for 30 min), and a final extension (72 °C for 8 min). The amplified PCR products together with a 100 bp DNA ladder (Thermo Scientific, Van Allen Way Carlsbad, CA, USA) were then size separated with 2% agarose gel electrophoresis.

#### 4.3.2. COLD1 (LOC_Os04g51180)

The tetra-primer amplification refractory mutation system (T-ARM)-PCR [10] was used to differentiate A/T mutation in *COLD1*, resulting in an allele-specific oligonucleotide PCR. The sequences surrounding the A/T variant were used to design T-ARM-PCR primers using the web-based program available from http://cedar.genetic.soton.ac.uk/public_html/primer1.htm l (accessed on 15 June 2019). Two pairs of primers (Table 1) were used to simultaneously amplify both alleles as well as an internal control DNA fragment in the same reaction. The A allele is indicated by amplification using an outer F primer and an inner R primer and the T allele is indicated by amplification using an inner F primer and an outer R primer control. These primers were first used to genotype two cold-tolerant cultivars, DPY and B30, and two cold-sensitive cultivars, SPR90 and RD 31, to determine polymorphism between them, and to obtain optimal PCR condition. Then, they were used to genotypes the 187 rice accessions. The genomic DNA samples were subjected to PCR amplification using two pairs of allele-specific primers. The PCR amplification was performed in 10 µL of reaction mixture, containing 1 µL of 10× Taq DNA polymerase buffer, 1 µL of 2 mM dNTP, 0.4 µL of 50 mM MgCl_2_, 0.1 µL of 5U/µL Taq DNA polymerase (Invitrogen, USA), 2 µL deionized water, 2 µL of 10–50 ng/µL DNA, 0.5 µL of 10 µM forward inner primer, 1 µL of 10 µM reverse inner primer, and 1 µL each of 1 µM forward outer primer and reverse outer primer. Thermal profiling was set with initial denaturation temperature of 95 °C for 5 min followed by 35 cycles of denaturation (95 °C for 30 s), annealing (57 °C for 30 s) and extension (72 °C for 30 min), and a final extension (72 °C for 8 min). The PCR products were separated by electrophoresis in 2% agarose gel. Finally, 100 bp DNA ladder (Thermo Scientific, USA) was used as a standard.

#### 4.3.3. LOC_Os10g34840

The sequences surrounding the G/A mutation in *LOC_Os10g34840* were used to design allele-specific primers using the web-based program available from http://cedar.genetic.soton.ac.uk/public_html/primer1.html (accessed on 15 January 2020). Two pairs of allele-specific primers were used to differentiate the alleles in separated reactions (Table 1). Similar to *COLD1*, first, these primers were used to genotype two cold-tolerant cultivars (DPY and B30) and two cold-sensitive cultivars (SPR90 and RD 31) to determine polymorphism between them, and to obtain optimal PCR condition. Then, they were used to genotype the 187 rice accessions. The PCR amplification was performed in 10 µL of reaction mixture, containing 1 µL of 10× Taq DNA polymerase buffer, 1 µL of 2 mM dNTP, 0.8 µL of 50 mM MgCl2, 0.1 µL of 5U/µL Taq DNA polymerase (Invitrogen, USA), 0.5 µL of DMSO, 2 µL of DNA, 3.1 µL deionized water, 2 µL of 10–50 ng/µL DNA, 0.5 µL of 1 µM of outer forward primers and 1 µL of 10 µM of inner reverse primers (for G allele), or 0.5 µL of 1 µM of outer reverse primers and 1 µL of 10 µM inner forward primers (for A allele). Thermal profiling was set with initial denaturation temperature of 95 °C for 5 min followed by 35 cycles of denaturation (95 °C for 30 s), annealing (70 °C for 30 s) and extension (72 °C for 30 min), and a final extension (72 °C for 8 min). The PCR products were separated by electrophoresis in 2% agarose gel. Finally, 100 bp DNA ladder (Thermo Scientific, USA) was used as a standard.

## 5. Conclusions

The cold tolerance under cold stress at the seedling stage was determined for 187 rice accessions. In addition, we developed 2 SNP functional markers from genes reported to be involved in cold tolerance, *COLD1* and *LOC_Os10g34840* and a novel indel marker in the *NAC6* gene. The *NAC6* marker was more sensitive than the other markers for predicting high cold tolerance. The genotypic assays developed in this study and the novel rice accessions with high cold tolerance will be useful for future rice breeding programs.

## Figures and Tables

**Table 1:** Gene-specific primers used in this study.

Gene	Sequence (5’–3’)	Tm °C	Allele	Size (bp)
*NAC6*	F-GCTTTGGGCCGCAGAAATTA	60	Tolerant	651
	R-TGTTGTAAATCCGGCACAGCA		Sensitive (deleted)	429
*COLD1*	Inner F-TCCTGGCTTACAGGGAAATTGATGAGAT	57	Control	320
	Inner R-AGCTGCCTTTCCAATGTTTTGATGTTCT		Tolerant (A)	225
	Outer F-AGTGTCATGGCTGTTCTTTCTGGTTTTG		Sensitive (T)	151
	Outer R-AGCACACCTCTTCTGATCCTTGAATCCT			
*LOC_Os10g34840*	Outer F-CGGCAGCGGAGGCGGTGGCTTC	70	Tolerant (G)	260
	Inner R-TCCCCGTGCAGGACGGCGCTCC			
	Inner F-CGACGGCGGGGACGGCGTCA	70	Sensitive (A)	203
	Outer R-GTCGCTGTCGCAGCCGCGCTGG			

## Data Availability

Not applicable.

## Supplementary Materials

The following supporting information can be downloaded at: https://www.mdpi.com/article/10.3390/plants12010215/s1, Table S1: Rice accessions used in the study, their agroecologies and special traits, subspecies, score for cold stress, genotyping using the 3 gene-specific markers, and survival rate.
