# Supplementary material for: Identification of Rice Accessions Having Cold Tolerance at the Seedling Stage and Development of Novel Genotypic Assays for Predicting Cold Tolerance
Source: Plants (Basel). 2023 Jan 3;12(1):215. doi: 10.3390/plants12010215 (PMC9823403; doi:10.3390/plants12010215)
Supplement: Supplementary file 1 [file plants-12-00215-s001.zip › Table S1 311022.pdf]

Table S1. Rice accessions used in the study, their agroecologies and special traits, subspecies, score for cold stress, genotyping using the 3 gene-specific markers, and survival rate

**GS. number**	**Rice accession**	**Agro-ecologies and special trait[b]**	**Subspecies[a,b]**	**score**	**Marker**				**Average survival rate±SE**
					NAC6	*COLD1*	Os10g0490100	3 marker	
1	Lueang Noi	Upland, Colored rice	Indica	1	N	N	N	N	0.00±0.00
2	E-GO	Upland, Colored rice	Indica	1	N	N	N	N	0.00±0.00
3	Daw Dawk Phrao	Upland rice	Indica	1	N	N	N	N	0.00±0.00
4	Khao Niaw Dam	Upland, Colored rice, Fragrance	Indica	1	N	N	N	N	8.82±8.82
5	OIC 27 III	Upland, Colored rice	Indica	1	N	N	N	N	13.66±7.77
6	Jam Pah-133	Upland rice	Indica	1	N	N	N	N	0.00±0.00
7	Jao Je Wah	Upland rice, South	Japonica	9	P	P	N	P2	100.00±0.00
8	Daw Muey	Upland, Colored rice	Indica	1	N	N	N	N	0.00±0.00
9	Jek Chuey	Upland rice	Indica	1	N	N	N	N	0.00±0.00
10	Ma Li Awng	Upland rice	Indica	1	N	N	N	N	0.00±0.00
12	Dawk Mai Khao B	nd	nd	1	N	N	N	N	2.94±2.94
13	Dawk Makh	Upland rice, North east	Admixture	2	N	N	N	N	22.69±7.31
14	Niaw Dam Hawm	Upland, Colored rice	Japonica	8	P	N	P	P2	89.16±4.95
15	Pong Tia	Upland rice	Indica	1	N	N	N	N	3.85±3.85
16	Khao Krabi	Upland rice	Indica	1	N	N	N	N	10.00±10.00
17	Khiaw Nok Kra Ling-2072	Upland rice	Indica	1	N	N	N	N	5.56±5.56
18	Ruang Diaw	Upland rice	Indica	2	P	N	N	P1	22.29±8.96
20	Gam	Upland, Colored rice	Indica	1	N	N	N	N	0.00±0.00
21	Glam	Upland, Colored rice	Indica	1	N	N	N	N	0.00±0.00
22	Gam	Upland, Colored rice	Indica	1	N	N	N	N	17.86±17.86
23	Khao Niaw Dam	Upland, Colored rice	Indica	1	N	N	N	N	2.50±2.50
24	Khiaw Nok Kra Ling-3578	Upland	Indica	1	N	N	N	N	18.38±6.62

26	Non Rai	Upland rice, Central	Indica	1	N	N	N	N	5.56±5.56
28	Khao Ma Led Lek	Upland rice	Indica	1	N	N	N	N	10.27±4.02
29	Khao Daeng	Upland, Colored rice	Indica	1	N	N	N	N	5.88±5.88
30	Mae Look Awn	Upland, Colored rice	Indica	3	N	N	N	N	31.11±8.89
31	Baw Den	Upland rice	Indica	1	N	N	N	N	16.32±1.32
32	Khao Klah	Upland rice	Indica	3	N	N	N	N	33.33±11.11
33	Chom Plee	Upland, Colored rice	Indica	3	N	N	N	N	38.89±11.11
36	Niaw Ga-Am-Mah-Ka	Upland, Colored rice	Admixture	1	N	N	N	N	11.54±11.54
37	Lueang Noi	Upland, Colored rice	Indica	3	N	N	N	N	39.12±7.54
38	Khao Gam	Upland, Colored rice	Indica	2	N	N	N	N	26.04±7.29
39	Khao Ruang Yao	Upland, Fragrance	Indica	1	N	N	N	N	2.63±2.63
40	GP 33 CAC 155	Upland, Colored rice	Indica	1	N	N	N	N	2.63±2.63
41	Khao Hawm	Upland rice	Indica	1	N	N	N	N	2.78±2.78
42	Ma Li Leuay	Upland rice	Indica	1	N	N	N	N	5.56±5.56
43	Tawng Mah Eng	Upland rice	Indica	1	N	N	N	N	14.17±5.83
44	Jam Pah Tawng Noi	Upland rice	Indica	1	N	N	N	N	0.00±0.00
45	Setti	Upland rice	Indica	1	N	N	N	N	7.69±7.69
46	Khao Sa Ahd	Upland rice	Indica	1	N	N	N	N	3.33±3.33
47	Plah Lai	Upland rice	Indica	3	N	N	N	N	35.59±5.59
48	Khao Piang	Upland rice	Indica	1	N	N	N	N	17.65±11.76
49	Gahb Dam	Upland rice	Indica	1	N	N	N	N	9.03±3.47
50	Daw Dam	Upland, Colored rice (dark purple stripe green leaves, dark purple stalk, brown speckle and black grain), and Fragrance	Indica	1	N	N	N	N	0.00±0.00
51	Khao Puang	Upland rice	Indica	1	N	N	N	N	0.00±0.00
52	Khao Khun Mae	Upland rice	Indica	1	N	N	N	N	5.00±5.00
53	Niaw Dam	Upland, Colored rice	Indica	1	N	N	N	N	0.00±0.00
54	Niaw Dam	Upland, Colored rice	Indica	1	N	N	N	N	0.00±0.00
55	Khao Tah Klueb	Upland rice	Indica	1	N	N	N	N	0.00±0.00

56	Niaw Dam	Upland, Colored rice	Indica	1	N	N	N	N	0.00±0.00
57	Khao Loi	Upland rice	Indica	1	N	N	N	N	0.00±0.00
58	Puang Tawng	Upland rice	Indica	1	N	N	N	N	0.00±0.00
59	Leuang	Upland rice	Indica	1	N	N	N	N	0.00±0.00
60	Niaw Dam Noi	Upland, Colored rice, Fragrance	Indica	1	N	N	N	N	0.00±0.00
61	Jao Hawm	Upland rice, North	Indica	1	N	N	N	N	0.00±0.00
62	Dawk Do	Upland, Colored rice	Indica	1	N	N	N	N	5.00±5.00
63	Niaw Pang Pah	Upland, Colored rice	Indica	1	N	N	N	N	12.50±12.50
65	Leuang Thong	Upland rice	Indica	1	N	N	N	N	0.00±0.00
66	Chao Kao	Upland rice, North east	Indica	8	N	N	N	N	84.87±9.87
67	Niaw Di Tah Ke	Upland, Colored rice	Indica	1	N	N	N	N	0.00±0.00
68	Nahng Prung	Upland rice	Indica	1	N	N	N	N	5.26±5.26
69	Tah Pib	Upland rice	Indica	1	N	N	N	N	0.00±0.00
70	Bueng Choo Bang Puey	Upland rice, Central	Japonica	9	P	N	N	P1	92.11±7.89
71	Toh Tia	Upland rice, Central	Japonica	9	P	N	P	P2	91.43±1.43
72	Bue Gi	Upland rice, Central	nd	9	P	N	P	P2	100.00±0.00
73	Pan Maw Wan	Upland rice, Central	Japonica	9	P	N	P	P2	100.00±0.00
74	Mee	Upland rice, Central	Indica	1	N	N	N	N	11.76±11.76
75	Ton Tia Guay Nu	Upland rice, Central	Japonica	9	P	N	P	P2	93.75±6.25
76	Pan Aung Jerng	Upland rice, Central	Indica	9	N	N	P	P1	93.75±6.25
77	Gra Bawk	Upland rice, North	Japonica	9	P	N	N	P1	92.11±7.89
79	Nang Khiaw	Upland, Colored rice	Indica	2	N	N	N	N	25.59±15.59
80	Leuang Thong	Upland, Colored rice	Indica	1	N	N	N	N	0.00±0.00
81	Khao Niaw	Upland rice	Indica	1	N	N	N	N	7.50±7.50
82	Niaw Lan Tan	Upland, Colored rice	Indica	3	N	N	N	N	35.00±10.00
83	Hae	Upland, Colored rice	Indica	9	N	N	N	N	90.63±9.38
84	Niaw Lan Tan	Upland, Colored rice	nd	1	N	N	N	N	10.00±10.00
85	Bue Lom	Upland, Colored rice	Indica	3	N	N	N	N	37.22±7.22
86	Niaw Look Pueng	Upland rice	Indica	6	N	N	N	N	63.82±11.18

87	Ja Pah Gaw	Upland, Colored rice	Indica	2	N	N	N	N	25.00±15.00
88	Pra Plenrg	Upland rice, Central	Indica	1	N	N	N	N	0.00±0.00
89	Jaek	Upland rice, South	Indica	5	N	N	N	N	56.03±8.97
90	Graw Saw	Upland, Colored rice, South	Indica	8	N	N	N	N	82.64±4.86
91	Chaw Mai	Upland, Colored rice, South	Indica	1	N	N	N	N	11.11±11.11
92	Sue Rah	Upland rice, South	Indica	1	N	N	N	N	0.00±0.00
93	Jao Tep Pah Rat	Upland rice, North east	Indica	1	N	N	N	N	2.50±2.50
94	Tah Gua Pah	Upland rice, North east	Indica	4	N	N	N	N	42.50±7.50
95	Huay Muang	Upland rice, Central	Japonica	9	N	N	P	P1	94.74±5.26
96	Lep Moo	Upland rice, Central	Indica	1	N	N	N	N	17.50±7.50
97	Sai Rai	Upland rice, South	Japonica	9	P	P	N	P2	100.00±0.00
98	Dawk Pud Rai	Upland rice, South	Japonica	9	P	P	N	P2	100.00±0.00
99	Jae San	Upland rice, South	Indica	1	N	N	N	N	15.79±15.79
101	Toh Mai	Upland rice, South	Indica	1	N	N	N	N	12.76±2.24
102	Ja Ge Wah	Upland rice, North	Indica	1	N	N	N	N	0.00±0.00
103	Ngaw Pin	Upland rice, North	Admixture	1	N	N	N	N	10.00±10.00
104	Gra Thing	Upland rice, Central	Indica	1	N	N	N	N	0.00±0.00
105	Ja Yee Ae	nd	nd	1	P	N	P	P2	7.50±2.50
107	Bue Kha Nong	Upland rice, Central	Indica	8	N	N	N	N	89.44±0.56
108	Jao Tah Hin Ngom	Upland rice, North east	Japonica	9	P	P	N	P2	97.37±2.63
109	E-Daeng	Upland rice, North east	Indica	1	N	N	N	N	0.00±0.00
110	E-Phae	Upland rice, North east	Indica	1	N	N	N	N	2.50±2.50
111	Dam Rai	Upland, Colored rice, South	Indica	1	N	N	N	N	5.00±5.00
112	Dam Hawm	Upland rice, South	Admixture	9	P	P	N	P2	100.00±0.00
114	Dawk Mud	Upland rice, South	Indica	6	N	N	N	N	65.00±15.00
115	Rice berry	Lowland, Breeding line	Indica	9	N	N	N	N	94.72±0.28
116	Gam Pe	Colored rice	Indica	1	N	N	N	N	0.00±0.00
117	Gam Ka	Colored rice	Indica	1	N	N	N	N	0.00±0.00
118	Gam Noi	Colored rice , Landrace rice in Northeast	nd	1	N	N	N	N	0.00±0.00

119	Gam Ton Khiaw	Colored rice	Indica	2	N	N	N	N	25.43±2.35
120	Hawm Nin-Plueak Muang	Lowland, Colored rice	Indica	1	N	N	N	N	5.00±5.00
121	Kahp Yao	Upland rice	Indica	1	N	N	N	N	12.50±12.50
122	Kai Noi Wiang Jan	Lowland rice	nd	8	N	N	N	N	87.50±2.50
123	Gam Piak Khahw	Upland rice	Indica	1	N	N	N	N	10.00±0.00
124	Khao Gon	Upland rice	Indica	9	N	N	N	N	100.00±0.00
125	Rathu	Resistance to Brown planthopper	Indica	2	N	N	N	N	30.00±10.00
126	TN1	High-yielding, semi-dwarf rice	Admixture	1	N	N	N	N	0.00±0.00
127	Khao Thua Dam	Upland rice	nd	1	N	N	N	N	0.00±0.00
128	Khao Kee chang	nd	nd	3	N	N	P	P1	37.50±17.50
129	Khao Thua Dam	Upland rice	Indica	1	N	N	N	N	0.00±0.00
130	Hawm Nin-Plueak Khao Ton Khiao	Lowland, Colored rice	Indica	1	N	N	N	N	13.89±13.89
131	Hawm Nin-caps	Lowland, Colored rice	Indica	1	N	N	N	N	5.00±5.00
132	Hawm Nin-132	Lowland, Colored rice	Indica	1	N	N	N	N	9.38±9.38
133	Sang Yod Thung Ku La	Lowland, Colored rice	Indica	1	N	N	N	N	5.26±5.26
134	Khao Niao Luam Pua	Colored rice , Landrace rice , Photosensitive	Indica	9	P	N	P	P2	93.75±6.23
135	Nipponbare (NB)	Temperate japonica	Japonica	9	P	P	P	P3	100.00±0.00
136	JAao Khao	Upland rice, North	Japonica	9	P	N	P	P2	92.50±7.50
137	Khao Niaw Dam	Upland, Colored rice	Indica	1	N	N	N	N	0.00±0.00
138	Khao Kad	nd	nd	1	N	N	N	N	0.00±0.00
139	Goo Mueang Luang	Upland rice, South, Resistance to Drought, Blast and Brown spot, Narrow brown spot	Indica	2	N	N	N	N	29.29±0.71
140	Nahm Sa Gui	Lowland, Rainfed for North and North East, Early flowering, Resistance to Brown planthopper	Indica	1	N	N	N	N	0.00±0.00
141	Kaen Jan	Landrace rice in South, Photosensitive	Indica	1	N	N	N	N	0.00±0.00
142	Lok Daeng Pat Ta nee	Landrace rice in South,Photosensitive	Indica	9	N	N	N	N	97.37±2.63

143	Pin Kaew 56	Landrace rice ,Photosensitive	Indica	1	N	N	N	N	0.00±0.00
144	Sang Yod	Lowland, Colored rice, Photosensitive	Indica	1	N	N	N	N	5.00±0.00
145	Khao Ngah Chang	Lowland rice, Resistance to salt	Indica	1	N	N	N	N	0.00±0.00
147	Khao Luang	Lowland rice, Photosensitive	nd	7	N	N	N	N	80.00±20.00
148	Kam Pai 15	Lowland rice, Photosensitive	nd	1	N	N	N	N	0.00±0.00
149	Khao Koo Deaw 35	Lowland, Rainfed for North ,Photosensitive	nd	8	N	N	N	N	89.61±5.39
150	Khao Ta Heang	Landrace rice in North,Photosensitive	Indica	4	N	N	N	N	50.00±7.89
151	Khem Thong Phat Tha Lung	Lowland rice, Photosensitive	Indica	2	N	N	N	N	25.39±9.61
152	Khai Mod Rin	Lowland rice, Photosensitive	Indica	1	N	N	N	N	19.01±3.22
153	Jek Chuey Kahp Khiao	Landrace rice in Central, Photosensitive	nd	1	N	N	N	N	7.50±7.50
154	Chum Phae 60	Breeding line, Photosensitive	nd	1	N	N	N	N	0.00±0.00
155	Chaw Lung 97	Lowland rice, Photosensitive ,South	nd	1	N	N	N	N	7.50±7.50
156	Nang Pa ya 132	Lowland rice, Photosensitive ,South	nd	1	N	N	N	N	7.50±7.50
157	Pat Ta Lung 60	Lowland rice, Photosensitive ,South	nd	2	N	N	N	N	28.03±6.97
158	Leb Nok Pattani	Lowland, Rainfed for South, Late flowering, Good cooking quality	Indica	2	N	N	N	N	30.00±15.00
159	Lang Gai	Upland, Colored rice	Indica	1	N	N	N	N	0.00±0.00
160	Hang Yee 71	Photosensitive	nd	1	N	N	N	N	7.50±7.50
161	Cho Mai Pai Pattani	landrace rice ,Photosensitive	nd	9	P	N	N	P1	93.75±6.25
162	Khao Luang San Pa Tong	nd	Admixture	1	N	N	N	N	2.63±2.63
163	Niao U Bon 2	Breeding line, Photosensitive, Northeast	nd	1	N	N	N	N	0.00±0.00
164	Lueang Pa Tiw 123	landrace rice ,Photosensitive , South	Indica	1	N	N	N	N	7.50±7.50
165	Gam Doi Sa Ked	Upland, Colored rice	Indica	1	N	N	N	N	0.00±0.00
166	Plai ngam Pra Gen Bu ree	landrace rice ,Photosensitive	Indica	2	N	N	N	N	21.25±8.75
167	R528	Upland rice, Non-photoperiod sensitivity Rice	nd	9	P	N	P	P2	97.50±2.50

168	Jao Li Saw San Pah Tong	Upland rice, North	Japonica	8	P	N	P	P2	83.33±16.67
169	Sew Mae Chan	Upland rice, North	Indica	5	N	N	N	N	54.09±1.46
170	Dawk Pah Yawm	Upland rice, South, Resistance to Blast, Brown spot, Narrow brown spot, Fragrance, Good cooking quality	Japonica	9	P	P	N	P2	100.00±0.00
172	KDML 105	Lowland, Breeding line, Photosensitive rainfed lowland rice, good eating quality, desirable fragrance	Indica	1	N	N	N	N	2.50±2.50
173	Leb Mea nang	landrace rice ,Photosensitive, Central	Indica	1	N	N	N	N	0.00±0.00
174	Chiang Phat Tha Lung	Lowland, Rainfed for South, Good milling quality	Indica	9	N	N	N	N	97.37±2.63
175	4806	Upland rice	Japonica	9	P	N	N	P1	100.00±0.00
176	PTT 1	Lowland, Improved line, Irrigated, Fragrance, High yielding	Indica	8	N	N	N	N	89.47±10.53
177	Azucena	Tropical japonica, Resistance to Drought	Japonica	8	P	N	N	P1	89.47±10.53
178	Suphan Buri 1	Breeding line, Non-photoperiod sensitivity Rice	Indica	7	N	N	N	N	73.24±3.24
179	Koshihikari	Temperate japonica	Japonica	9	P	P	P	P3	93.75±6.25
180	PSL60-2	Breeding line, Non-photoperiod sensitivity Rice	Indica	1	P	N	N	P1	10.00±10.00
181	Hawm Nin	Colored rice, Breeding line, Non-photoperiod sensitivity Rice	Indica	9	N	N	N	N	92.50±7.50
182	Pin Ka sat 3	Breeding line, Non-photoperiod sensitivity Rice	Indica	5	N	N	N	N	52.50±7.50
183	Khao' Yipun DOA	Japonica, Non-photoperiod sensitivity Rice	Japonica	9	P	P	P	P3	100.00±0.00
184	B11	Breeding line, Indica	Indica	8	N	N	N	N	86.55±2.34
185	B29	Breeding line, Indica	Indica	7	N	N	N	N	70.83±4.17
186	B30	Tropical japonica	Japonica	9	P	N	P	P2	96.43±3.57
187	B61	Breeding line, Indica	Indica	9	N	N	N	N	100.00±0.00
188	RD7	Breeding line, Irriqated lowland Rice ,Non-photoperiod sensitivity Rice	Indica	8	P	N	N	P1	81.25±18.75

189	RD29	Breeding line, Irriqated lowland Rice ,Non-photoperiod sensitivity Rice	Indica	2	N	N	N	N	26.39±1.39
190	RD31	Breeding line, Irriqated lowland Rice ,Non-photoperiod sensitivity Rice	Indica	1	N	N	N	N	20.00±10.00
191	RD39	Lowland, Improved line, Non-photosensitive, Resistance to Blast, good cooking quality	Indica	9	N	N	N	N	97.06±2.94
192	RD41	Breeding line, Irriqated lowland Rice ,Non-photoperiod sensitivity Rice	Indica	5	N	N	N	N	57.35±7.35
193	RD47	Breeding line, Irriqated lowland Rice ,Non-photoperiod sensitivity Rice	Indica	3	N	N	N	N	37.65±2.35
194	Phitsanulok 2	Breeding line, Irriqated lowland Rice ,Non-photoperiod sensitivity Rice	Indica	7	N	N	N	N	75.00±8.33
195	Chai Nat 1	Breeding line ,Non-photoperiod sensitivity Rice	Indica	1	N	N	N	N	18.75±18.75
196	Suphan Buri 60	Breeding line ,Non-photoperiod sensitivity Rice	Indica	9	N	N	N	N	92.11±7.89
197	Suphan Buri 90	Breeding line ,Non-photoperiod sensitivity Rice	Indica	1	N	N	N	N	12.94±7.06
198	RD23	Breeding line, Irriqated lowland Rice ,Non-photoperiod sensitivity Rice	Indica	8	N	N	N	N	88.89±11.11
199	Hawm Chon La sit	Breeding line ,Non-photoperiod sensitivity Rice	Indica	8	N	N	N	N	88.89±11.11
200	Khao Jow Hawm Suphan Buri	Breeding line, Irriqated lowland Rice ,Non-photoperiod sensitivity Rice	Indica	8	N	N	N	N	89.38±4.38

1=Survival rate average <20%

2=Survival rate average21-30%

3=Survival rate average 31-40%

4=Survival rate average 41-50%

5=Survival rate average 51-60%

6=Survival rate average 61-70%

7=Survival rate average 71-80%

8=Survival rate average 81-90%

9=Survival rate average >90%

P3 =with 3 marker

P2 =with 2 marker

P1 =with 1 marker
